# Generation of Trauma Rush Call in Managing Patients With Road Traffic Injuries Visiting the Emergency Department: A Retrospective Analysis

**DOI:** 10.7759/cureus.75564

**Published:** 2024-12-11

**Authors:** Nazir Najeeb Kapadia, Salman M Soomar, Badar Afzal, Emaduddin Siddiqui

**Affiliations:** 1 Emergency Medicine Department, King Hamad University Hospital, Busaiteen, BHR; 2 Emergency Medicine Department, Aga Khan University, Karachi, PAK; 3 Emergency Department, King's College Hospital London, Dubai, ARE

**Keywords:** emergency, public health, road traffic injuries, trauma, trauma rush call

## Abstract

Background: Road traffic injuries (RTIs) are currently the ninth most common cause of mortality and are expected to increase in the future. RTIs rank in the top three reasons why young people die. Because of the high incidence and mortality risk, proper trauma care has been prioritized for RTI patients who present to the emergency department. Making appropriate and prompt trauma emergency calls can save lives. Poor resource use and an increased burden of inquiry can result from the inappropriate development of trauma rush calls (TRCs). Therefore, this study aims to evaluate the appropriate generation of TRCs to manage patients with RTIs presenting to the emergency department (ED).

Methods: A retrospective analysis was conducted at the ED of the private tertiary care hospital in Karachi, Pakistan. Patients with RTIs and TRCs generated for the management were included. Dead on arrival was excluded. A total of 322 patients were enrolled using convenient sampling. A standard Performa was developed to collect the data from medical records, including demographics, clinical characteristics, radiological findings, TRC characteristics, and disposition. Multivariable logistic regression analysis was applied to determine the TRC justified using odds ratios, considering a p-value ≤0.05 as significant.

Results: Of the total 322 patients, the majority were males (n=266, 82.61%) between 21 and 40 years of age. Most patients reported injury/accident through high-speed vehicle crash (n=102, 31.68%). A Glasgow Coma Scale (GCS) score of <14 was recorded in 119 (36.96%) patients, and of 208 patients, 153 (73.55%) had positive computed tomography (CT) head findings, majorly subdural hematoma (n=43, 28.10%). CT cervical spine showed fractures in 14 patients (11.29%). Only one patient had both a positive CT abdomen and focused assessment with sonography in trauma (FAST), with a low systolic blood pressure (SBP) of 85 mmHg, and the CT abdomen showed a grade 3 spleen laceration. TRC was justified in 248 (77.01%) patients. Multivariable analysis showed that a GCS <14 [adjusted odds ratio (aOR) 3.03 (95% CI: 2.58-5.04)] and cervical collar [aOR 3.12 (95% CI: 1.63-5.98)] were the significant predictors for the TRC justified.

Conclusion: A GCS <14 and cervical collar significantly justify the generation of TRC. These findings are crucial in educating ED staff regarding optimal care delivery. The future deployment of resources depends on these findings for skill development.

## Introduction

Road traffic injuries (RTIs) are one of the major public health problems in many countries worldwide, particularly in low- and middle-income countries (LMICs) [1-3]. Globally, RTI is the eighth leading cause of death, and it is projected to rise to the top five by 2030 [4,5]. RTIs are among the top three causes of death, in addition to being the leading cause of death for young people aged between 5 and 29 years [3].

Over 90% of the annual 1.35 million worldwide deaths are due to RTIs occurring in LMICs [6]. Death rates attributable to RTIs in LMICs are more than double those in high-income countries (HICs) [3]. Furthermore, the situation is even worse in rural areas, as corroborated by a study in Ghana, which found that 61.2% of road traffic fatalities and 52.3% of RTIs occurred on roads in rural areas [3-5]. A study from Pakistan found that the risk for RTIs was higher in males and in those aged between 16 and 45 years [6]. An Indian study reported that 64.8% of RTI deaths occurred in males between 15 and 59 years of age [7]. Another study from Bangladesh found that in patients with RTIs, the most injured age group was 20-35 years old, and 76% of patients were male [3]. Similarly, a Nepalese study noted that most of the RTIs were found in males aged 20-40 years [2]. Rates for RTIs in Ghana and Uganda are higher than those reported in Pakistan [6]. 

It is important to promptly manage trauma patients. The "golden hour" concept emphasizes the increased risk of death and the need for rapid intervention during the first hour of care following major trauma [8]. Undoubtedly, there are instances when rapid intervention improves the outcome of injured patients (e.g., obstructed airway, tension pneumothorax, severe hemorrhage) [9].

With recent advancements in the field of emergency medicine, trauma care has been emphasized due to the high risk of incidence and mortality. Advanced Trauma Life Support (ATLS) is a program for healthcare providers to manage acute trauma cases, developed by the American College of Surgeons [10]. Retrospective research analyzing mortality rates in the pre-ATLS versus post-ATLS eras has revealed a 20% drop at one large international institution and a statistically significant reduction in deaths within the first hour at two US community hospitals [11]. Existing literature records that the ATLS played a role in increasing nurses’ knowledge regarding trauma triage [12]. An Iranian study showed that the ATLS had a significant role in improving the clinical knowledge and practical skills of emergency room nurses in trauma care [13]. ATLS provides a structured and efficient approach to the management of patients presenting with trauma and RTIs in the emergency department (ED) [10]. Several factors should drive protocols for trauma team activation (TTA), including defining the severely injured patients and determining the immediate resources needed to deliver optimal care to the patient [14]. The performance of a well-functioning interdisciplinary trauma team coordinated by an experienced trauma leader plays a pivotal role during the initial phase of patient care for patients who are severely injured. A trauma team comprises general surgery, neurosurgery, orthopedics, radiology, and anesthesia teams. The appropriate management of severely injured patients with trauma/RTIs depends on the generation of trauma rush calls (TRCs) [15,16]. The primary goal of the trauma team is to ensure that the resources necessary to address the clinical needs of injured patients are immediately available and utilized by carrying out relevant interventions required in a timely fashion [17].

It has been seen that inappropriate generation of TRCs, based on the criteria of physician discretion, can lead to an increased burden of investigation and poor utilization of resources [16]. In LMICs where patients have to pay out of their pockets, it has a great financial burden. RTIs have a notable influence on the national economy as LMICs lose approximately 5% of their GDP due to RTIs, as opposed to 3% for the rest of the world [3]. RTIs also significantly affect the health-related quality of life as they have physical, psychological, emotional, and monetary ramifications [3]. A delay in the activation of the rapid response team is linked to increased mortality and worse morbidity outcomes as well as increased length of stay (LOS) in the hospital [18].

Having both low and high thresholds on the activation of TRCs can either compromise a patient’s finances or health, respectively. The standards for TTA require modifications in accordance with local caseload and injury trends, which are prone to differ across various geographical areas [19]. The predictive properties of triage criteria depend on the prevalence and spectrum of severe injuries [19]. Trauma-informed care best practices can improve patient care by informing patient-provider communication in the ED, medical education, department leadership, and organizational structure, in addition to fostering safer spaces for ED patients and healthcare workers by creating more thoughtful, therapeutic relationships with patients [20]. Therefore, this study aims to evaluate the appropriate generation of TRCs to manage patients with RTIs visiting the ED.

## Materials and methods

Operational definition

Trauma Rush Call

The activation of a group of teams comprising general surgery, orthopedics, neurosurgery, and emergency physicians to manage severe trauma (Table 5).

Study design and setting

A retrospective analysis was conducted at the ED of the private tertiary care hospital in Karachi, Pakistan.

Study duration

The study was conducted from August 1 to December 31, 2020. 

Study participants and eligibility criteria

All patients of pediatrics and adult age groups coming with RTIs and TRC were generated for the management. Patients who were dead on arrival (DOA) were excluded.

Sampling technique and sample size

A non-probability convenient sampling method was used to enroll the participants. A total of 322 patients were enrolled in the study.

Ethical approval

Ethical approval was obtained from the Ethics Review Committee of Aga Khan University Hospital, Karachi, Pakistan (ERC Ref. no. 4298-EM-ERC-16). This research was conducted in accordance with the Declaration of Helsinki. Written and verbal informed consent was waived by the Ethical Review Committee of The Aga Khan University Hospital, Karachi, Pakistan.

Data collection and management

Medical record files were reviewed and analyzed for their indication of TRCs. A standard Performa was developed to collect the data, including demographics, clinical characteristics, radiological findings, TRC characteristics, and disposition. Patients were labeled as TRC justified based on retrospective data, which were collected by data collectors and reviewed by an ATLS provider-trained emergency physician who was not part of this study just to exclude the bias. As per the ATLS guidelines, the patients were marked as yes or no for TRC justified upon review of the various variables and the factors included in the data. The collected data were transferred to statistical software where the data were checked for missing values.

Statistical analysis

Normally distributed quantitative continuous data were summarized as mean and standard deviations (SD), while skewed data were presented as median and interquartile range (IQR). Categorical variables were presented as frequencies and percentages. Data were stratified on the rush call justified (yes/no). A post-stratification chi-square test was applied. Multivariable logistic regression was also applied to determine the crude odds ratio (cOR) and adjusted odds ratio (aOR), considering a p-value ≤0.05 as significant.

## Results

Of the total 322 patients, the majority were male (n=266, 82.61%) between 21 and 40 years old with a mean± SD of 32.79±16.73. A few of the patients reported comorbidities, including diabetes and hypertension (Table 1).

**Table 1:** Baseline and clinical characteristics of the patients. CKD: chronic kidney disease.

Characteristics	n (%)
Age (mean ±SD)	32.79 ±16.73
Age	
1-20	77 (23.91)
21-40	153 (47.52)
41-60	75 (23.29)
>60	17 (5.28)
Gender	
Male	266 (82.61)
Female	56 (17.39)
Comorbidities	
Diabetes	11 (3.42)
Hypertension	10 (3.11)
Ischemic heart disease	2 (0.62)
CKD	1 (0.31)

Most patients reported injury/accident through high-speed vehicle crash (n=102, 31.68%); the majority reported the severity as penetrating injury (n=75, 23.29%). The median (IQR) systolic blood pressure (SBP) of patients was 121 mmHg (107-135) and oxygen saturation was 98% (97-100). The majority of patients reported bilateral (BL) air entry (n=284, 88.20%). However, 119 (36.96%) had a GCS score <14, and computed tomography (CT) head was done for 208 patients (64.60%). Of 208, 153 patients (73.55%) had positive CT head findings, majorly subdural hematoma (n=43, 28.10%). CT cervical spine was done for 124 patients (38.63%), with most showing normal findings; 14 (11.29%) showed fractures. Cervical collars were given to 181 patients (56.21%) with positive CT head and cervical findings. CT abdomen was done for 80 patients (24.92%), of which 51 were positive. Only one patient had both a positive CT abdomen and focused assessment with sonography in trauma (FAST) with a low SBP of 85 mmHg, and the CT abdomen showed a grade 3 spleen laceration. Chest X-ray was normal for 225 patients (70.53%), and FAST was negative for 225 patients (69.88) (Table 2).

**Table 2:** Injury characteristics and rush call determinants of the patients. SBP: systolic blood pressure; GCS: Glasgow Coma Scale; CT: computed tomography; FAST: focused assessment with sonography in trauma; SCU: special care unit; ICU: intensive care unit; CTS: cardiothoracic surgery.

Characteristics	n (%)
Severity of injury	
Penetrating injury	75 (23.29)
Evidence of spinal cord injury	12 (3.73)
Open or depressed skull fracture	4 (1.24)
Two or more long bone fractures	10 (3.11)
Crush or degloving injury	16 (4.97)
Pelvic fracture	11 (3.42)
Flail chest	11 (3.42)
Mechanism of injury	
Ejection from automobile	8 (2.48)
Fall >20 feet, >10 feet (children) or >2-3 times the height	21 (6.52)
High speed vehicle crash	102 (31.68)
Auto vs. pedestrian >5 m/hour	23 (7.14)
Bike >20 m/hour	84 (26.09)
Roll over	30 (9.32)
Gunshot	22 (6.83)
Blast victim	16 (4.97)
Others	16 (4.97)
SBP (median, IQR)	121 mmHg (107-135)
SBP <90	18 (5.59)
Oxygen saturation (SpO_2_) (median, IQR)	98% (97-100)
BL air entry	
Yes	284 (88.20)
No	38 (11.80)
GCS (median, IQR)	15 (13-15)
GCS <14	119 (36.96)
CT head	
Yes	208 (64.60)
No	114 (35.40)
Cervical collar	
Yes	181 (56.21)
No	141 (43.79)
CT cervical spine	
Yes	124 (38.63)
No	197 (61.37)
Cervical tenderness	
Yes	17 (5.28)
No	305 (94.72)
CT relevant	
Yes	97 (30.22)
No	224 (69.78)
Chest X-ray	
Not done	9 (2.82)
Normal	225 (70.53)
Fracture	26 (8.15)
Hemothorax	14 (4.39)
Pneumothorax	27 (8.46)
Others	18 (5.64)
FAST	
Positive	16 (4.97)
Negative	225 (69.88)
Disposition service	
General surgery (trauma team)	83 (25.86)
Orthopedics	57 (17.76)
Neurosurgery	130 (40.50)
CTS	15 (4.67)
Plastic surgery	17 (5.30)
Pediatric surgery	14 (4.36)
Others	5 (1.55)
Disposition care	
Ward	134 (41.61)
SCU	142 (44.10)
ICU	46 (14.29)

Of the total 322 patients, rush call was justified in 248 (77.01%). TRC was significantly justified for patients with a GCS <14 (n=87, 35.08%), CT head (n=175, 70.56%), and CT cervical (n=114, 46.15%). Furthermore, rush call was justified in patients with cervical collar (n=162, 65.32%). Patients who were disposed to neurosurgery (n=119, 48.18%) and general surgery (trauma team) (n=67, 27.13%) justified the rush call (Table 3).

**Table 3:** Injury characteristics and determinants stratified on TRC justified. ^*^Fisher’s exact test. FAST: focused assessment with sonography in trauma; GS: general surgery; TRC: trauma rush call; SCU: special care unit; ICU: intensive care unit; BL: bilateral; CTS: cardiothoracic surgery.

Characteristics	TRC justified		p-value
	Yes (248) n (%)	No (74) n (%)	
Severity of injury*			
Penetrating injury	52 (20.97)	23 (31.08)	0.528
Evidence of spinal cord injury	11 (4.44)	1 (1.35)	
Open or depressed skull fracture	4 (1.61)	0 (0.00)	
Two or more long bone fractures	8 (3.23)	2 (2.70)	
Crush or degloving injury	12 (4.84)	4 (5.41)	
Pelvic fracture	9 (3.63)	2 (2.70)	
Flail chest	9 (3.63)	2 (2.70)	
Mechanism of injury*			
Ejection from automobile	6 (2.42)	2 (2.70)	0.755
Fall >20 feet, >10 feet (children) or >2-3 times the height	16 (6.45)	5 (6.76)	
High speed vehicle crash	75 (30.24)	27 (36.49)	
Auto vs. pedestrian >5 m/hour	16 (6.45)	7 (9.46)	
Bike >20 m/hour	70 (28.23)	14 (18.92)	
Roll over	25 (10.08)	5 (6.76)	
Gunshot	16 (6.45)	6 (8.11)	
Blast victim	13 (5.24)	3 (4.05)	
Others	11 (4.44)	5 (6.76)	
SBP <90*	16 (6.45)	2 (2.70)	0.218
SpO_2_ <98	92 (37.10)	16 (21.62)	0.013
BL air entry			
Yes	212 (85.48)	72 (97.30)	0.006
GCS <14*	87 (35.08)	1 (1.35)	<0.001
CT head			
Yes	175 (70.56)	33 (44.59)	<0.001
Cervical collar			
Yes	162 (65.32)	19 (25.68)	<0.001
CT cervical spine			
Yes	114 (46.15)	10 (13.51)	<0.001
Cervical tenderness*			
Yes	17 (6.85)	0 (0.00)	0.021
Chest X-ray			
Not done	4 (1.62)	5 (6.94)	0.022
Normal	169 (68.42)	56 (77.78)	
Fracture	20 (8.10)	6 (8.33)	
Hemothorax	13 (5.26)	1 (1.39)	
Pneumothorax	25 (10.12)	2 (2.78)	
Others	16 (6.48)	2 (2.78)	
FAST			
Negative	164 (66.13)	61 (82.43)	0.01
Disposition service			
GS (trauma team)	67 (27.13)	16 (21.62)	<0.001
Orthopedics	23 (9.31)	34 (45.95)	
Neurosurgery	119 (48.18)	11 (14.86)	
CTS	14 (5.67)	1 (1.35)	
Plastic surgery	9 (3.64)	8 (10.81)	
Pediatric surgery	12 (4.86)	2 (2.70)	
Others	3 (1.21)	2 (2.70)	
Disposition care*			
Ward	63 (25.40)	71 (95.95)	<0.001
SCU	140 (56.45)	2 (2.70)	
ICU	45 (18.15)	1 (1.35)	

CT scan findings showed that the majority of the subdural hematoma (24.24%) and extradural hematoma (18.18%) patients had a GCS <14. Additionally, 20.0% of these patients had blood pressure (SBP <90).

Patients with penetrating injury (30.77%) and spinal cord injury (53.85) had positive findings on CT cervical spine. Only six (6.82) patients with cervical tenderness had a GCS <14, while 11 (47.83%) had positive findings on CT cervical spine. The majority of patients with low GCS were disposed to the special care unit (SCU) (46, 52.27), followed by the intensive care unit (ICU) (30, 34.09) (Table 6).

Multivariable analysis showed that a GCS <14 [3.03 (95% CI: 2.58-5.04), p<0.001] and cervical collar [3.12 (95% CI: 1.63-5.98), p=0.001] were the significant predictors for the TRC justified. However, BL air entry and FAST negative were the protective characteristics for TRC (Table 4).

**Table 4:** Multivariable analysis reporting cOR and aOR with 95% confidence interval. ^*^Significant at univariate level. ^**^Significant at multivariable level. CKD: chronic kidney disease; CT: computed tomography; FAST: focused assessment with sonography in trauma; BL: bilateral; cOR: crude odds ratio; aOR: adjusted odds ratio.

Characteristic	cOR	p-value	aOR	p-value
Age				
1-20	1	0.309	-	-
21-40	1.66 (0.67-3.55)		-	
41-60	1.15 (0.45-3.29)		-	
>60	1.58 (0.53-3.40)		-	
Gender				
Male	1.59 (1.29-4.05)	0.005*	1.10 (1.05-2.31)	0.001**
Comorbidities			-	
Diabetes	1	0.377	-	-
Hypertension	2.6 (1.37-7.06)		-	
Ischemic heart disease	1.25 (0.47-6.43)		-	
CKD	1.83 (0.73-4.65)		-	
Severity of Injury			-	
Penetrating injury	1	0.525	-	-
Evidence of spinal cord injury	4.86 (0.59-9.93)		-	
Open or depressed skull fracture	2.26 (1.38-3.69)		-	
Two or more long bone fractures	1.76 (0.34-4.98)		-	
Crush or degloving injury	1.32 (0.38-4.55)		-	
Pelvic fracture	1.99 (0.39-4.94)		-	
Flail chest	1.63 (0.33-4.96)		-	
Mechanism of injury				
Ejection from automobile	1	0.745	-	-
Fall >20 feet, >10 feet (children) or >2-3 times the height	1.06 (0.78)		-	
High speed vehicle crash	1.92 (0.92-4.86)		-	
Auto vs. pedestrian >5 m/hour	1.66 (0.77-4.75)		-	
Bike >20 m/hour	1.88 (0.90-5.68)		-	
Roll over	1.44 (0.72-4.99)		-	
Gunshot	1.49 (0.75-4.42)		-	
Blast victim	1.58 (0.55-5.56)		-	
Others	1.02 (0.71-4.99)		-	
SBP <90	2.48 (0.55-4.19)	0.481	-	-
SpO_2_ <98	2.13 (1.16-3.93)	0.011*	0.90 (0.43-1.90)	0.798
BL air entry				
Yes	0.69 (0.16-0.98)	0.001*	0.37 (0.17-0.78)	0.023*
GCS <14	3.94 (1.67-5.38)	<0.001*	3.03 (2.58-5.04)	<0.001**
CT head				
Yes	2.97 (1.74-5.07)	0.001*	1.65 (0.95-2.89)	0.075
Cervical collar				
Yes	1.56 (1.26-3.04)	<0.001*	3.12 (1.63-5.98)	0.001**
CT cervical spine				
Yes	2.07 (1.54-2.80)	<0.001*	1.79 (0.69-4.63)	0.224
Cervical tenderness				
Yes	3.12 (2.40-4.05)	<0.001*	1.41 (0.99-1.99)	0.051
Chest X-ray				
Normal	0.90 (0.75-1.97)	0.277	-	-
Fracture	3.89 (1.52-4.19)		-	
Hemothorax	3.75 (1.20-4.06)		-	
Pneumothorax	3.33 (1.18-4.25)		-	
Others	2.4 (1.02-4.19)		-	
FAST				
Negative	0.51 (0.26-0.99)	0.039*	0.33 (0.15-0.69)	0.003**

## Discussion

This study presents several noteworthy findings that investigate the factors justifying TRC for patients with RTIs presenting to the ED. TRC was justified in a substantial percentage of patients, specifically 77.01% (n=248). Most of the patients, precisely 31.68% (n=102), suffered injuries through high-speed vehicle crashes, while 23.29% (n=75) reported penetrating injuries. It details typical situations in which the trauma team is activated, consisting of a traumatic event and a GCS <14. Here, traumatic events include RTI by high-speed collision (>30 mph); penetrating injuries to the head, neck, chest, abdomen, and pelvis; and open or depressed skull fracture, pelvic fracture, two or more proximal long bone fractures, and flail chest.

Our multivariate analysis concluded that male gender, GCS <14, and cervical collar were significant predictors for the TRCs justified. Most of the patients were male, accounting for 82.61% (n=22), and aged between 21 and 40 years. Similarly, in an Ethiopian study, Getachew et al. reported that RTI-associated ED admissions are most prevalent in males and those aged 25-44 years [21]. A study from Nigeria by Ibrahim et al. reported the same, noting that 72% of trauma patients due to RTI were males, and the mean age was 33.5 years [22]. Furthermore, a study from Pakistan found that 81.3% of all road traffic accident cases were male, and the mean age of the patients involved was 28.2 years, further detailing that only 8.1% of patients reached the hospital within the golden hour [23].

Alongside GCS<14, other significant predictors of TRCs found in our study include patients requiring CT head, CT cervical spine, and those with a cervical collar. From a clinical perspective, the GCS is a crucial neurological criterion, particularly in head trauma (traumatic brain injury), for which the ATLS supports its use to gauge the severity of the injury [18]. Norwood et al. stated that the use of a GCS score of 14 for trauma team activation would have amended their under-triage rate from 19.1% to 4.4%, thereby concluding that a GCS ≤14 correctly anticipates the need for full activation of a trauma team and patient admissions after motor vehicle accidents [19]. Hence, it allows for the correct assessment of these patients in case of any overlooked injuries that may develop into a neurosurgical emergency [18].

This is in line with our finding that CT head scans also significantly justified TRCs, as 1.24% (n=4) of patients suffered from open or depressed skull fractures. In addition, CT cervical spine scans required for 3.73% (n=12) of patients showing evidence of spinal cord injury are also linked to the use of cervical collars. Patients suffering from two or more long bone fractures constituted 3.11% (n=10). In contrast, Getachew et al. recorded that lower limb injuries (36%) occurred most commonly, followed by head injuries (20%), while the study from Pakistan also stated that lower extremity injuries occurred most commonly (44.7%), followed by head and neck injuries (27.8%) [21,23].

However, 3.42% (n=11) suffered a pelvic fracture and flail chest; chest X-ray was normal and FAST was negative in the majority of patients. The categorization of BL air entry and FAST negative as “protective characteristics” is indicative of its lesser role in the generation of rush calls. On the contrary, existing literature shows that in China RTIs continue to remain the main causative factor for thoracic injuries [20].

The rush call was also justified in patients who were disposed to Neurosurgery and GS (trauma team). Emergency physicians and general surgeons on trauma resuscitation teams are essential, in addition to neurosurgeons and orthopedic surgeons for specialized care [24]. A study conducted in the United Kingdom (UK) found that of all the trauma calls, most noted road traffic accidents as the mechanism of injury, and the majority of patients experienced orthopedic and/or neurosurgical injuries [25]. A study from New Delhi, India, based in the ED of Neurosurgery, found that of all the patients with mild traumatic brain injury, RTIs were the main causative factor (50.6%) [26]. 

In contrast to our study, Dattani et al. stated that after the early assessment in the ED, patients undergo immediate transfer to orthopedic surgeons, and even though general surgeons examined 30% of the patients, merely a few were admitted under their care [25]. Therefore, concluding that the majority of patients did not require general surgical intervention, even though the availability of a general surgeon was essential in those with penetrating injuries [25]. Conversely, an Ethiopian study found that the ED outcome of RTIs involved 78% of cases in need of hospital admission, of which 62% and 16% were hospitalized in the surgical and orthopedic departments, respectively [21].

As corroborated by our findings, guidelines by the Committee on Trauma of the American College of Surgeons correctly requisite the initial involvement of general surgeons in trauma team activations at all three levels of trauma center verification. The availability of the general surgeon in the ED is required for major resuscitations and to facilitate the prompt treatment of critically injured patients [19].

Establishing accurate thresholds for the justified generation of TRC is crucial for the delivery of appropriate trauma care and in ensuring that patients have the optimal quality of life post trauma. Prompt activation of the trauma team directly affects outcomes for patients [27]. A study from Florida, USA, found that triage to a trauma center is linked to an 18% improvement in survival, leading to a marginal cost per life saved of $746 to $2815, which is advantageous compared to societal expenses for other health issues [28]. Hence, demonstrating that the evaluation of the financial efficiency of a trauma system is dependent on the restoration of trauma victims to a productive life [28]. A study from China also concluded that in patients who had moderate chances of survival, undercall is linked to reduced survival [26]. It further detailed that of all the patients who met the criteria for a trauma call, 72% of them correctly triggered the trauma call [26].

Injury is accountable for a rising global burden of death and disability [27]. Interpretation of data from the Global Burden of Disease indicates that almost 2 million lives can be saved annually if the death rates of seriously injured people in LMICs resemble those achieved in HIC [28]. In the USA, 34% of patients are undertriaged. A quality improvement assessment in a Canadian center found that they had high levels of undertriage [27]. Their trauma team activation compliance rates were less than 60% and were improved to over 90%, in accordance with Accreditation Canada guidelines [27].

Evaluations in LMICs identify limited resources as a barrier to providing sufficient care for the injured [28]. However, some contextual recommendations are also present to assist policymakers and planners in LMICs to tailor trauma care systems and set priorities in these resource-limited settings [28]. Organizing systems for trauma management (e.g. protocols to appoint trauma centers, prehospital triage protocols, and transfer specifications) and verification and accreditation of trauma care services are some of the existing evidence from HICs covering organizational and administrative characteristics that can be incorporated in resource-constrained settings [28].

Strengths and limitations

The strengths of this study include the large sample size used and a robust methodology and analysis, focusing specifically on trauma due to RTIs. A range of variables are used, including demographic, injury, and clinical characteristics. The use of a multivariate analysis assists in identifying independent determinants of TRC, which further assists in deducing the impact of multiple factors.

The limitations include the retrospective design of the study, which contributes to a potential risk for bias and inability to establish causation. This study was conducted in a single-center tertiary care hospital, which is responsible for limiting the generalizability of these findings to other settings. The use of a control group that did not trigger TRCs prevents the comparison of any outcomes or impact of the TRC.

Recommendations

TRC training should be included in the residency program and medical education to better understand when the TRC is generated and what measures should be considered. This will help save lives and avoid burnout in the physicians who indulge in false TRC.

## Conclusions

GCS <14, CT head, CT spine, cervical collar, and disposition to Neurosurgery and GS significantly justify the generation of TRC. This is in contrast to BL air entry and negative FAST exams, which suggest a lower probability of TRC. These findings are crucial in educating ED staff regarding optimal care delivery. The future deployment of resources, time, and care is dependent on these findings, particularly in LMICs that have limited resources and skill development. Additionally, future research is essential to further expedite and enhance patient care and refine resource utilization.

## Appendices

**Table 5:** Criteria for trauma team activation. BSA: body surface area; GCS: Glasgow Coma Scale.

Category	Criteria
Mechanism of Injury	Falls >5 m (16.5 feet) High-speed motor vehicle accident Ejection from vehicle High-speed motor vehicle collision Pedestrian, bicyclist, or motorcyclist vs. vehicle >30 kph (18 mph) Fatality in the same vehicle
Specific Injuries	Injury to more than two body regions Penetrating injury to the head, neck, torso, or proximal limb Amputation Burn >15% BSA adults, 10% BSA children or involving airway Airway obstruction
Physiological Derangement	Systolic <90 mmHg Pulse >130 RR <10 or >30 GCS score <14/15 Chest injury in patient older than 70 years Pregnancy >24 weeks with torso injury

**Table 6:** Association of different determinants in TRC. CT: computed tomography; TRC: trauma rush call; GCS: Glasgow Coma Scale; SAH: subarachnoid hemorrhage; SDH: subdural hemorrhage; EDH: epidural hemorrhage; DAI: diffuse axonal injury; IVH: intraventricular hemorrhage; SBP: systolic blood pressure; FAST: focused assessment with sonography in trauma; SCU: special care unit; ICU: intensive care unit.

Characteristic	Rush call determinant	P-value
Positive CT findings	GCS <14	
SAH	5 (7.58)	0.066
SDH	16 (24.24)	
EDH	12 (18.18)	
DAI	4 (6.06)	
Fractures	3 (4.55)	
Contusions	8 (12.12)	
Cerebral edema	1 (1.52)	
IVH	2 (3.03)	
Other specific injuries	3 (4.55)	
Positive CT findings	SBP <90	
SAH	2 (13.33)	0.817
SDH	3 (20.0)	
EDH	3 (20.0)	
DAI	1 (6.67)	
Fractures	0 (0.0)	
Contusions	1 (6.67)	
Cerebral edema	0 (0.0)	
IVH	0 (0.0)	
Other specific injuries	1 (6.67)	
CT cervical spine	Cervical tenderness	
Normal	6 (85.71)	0.990
Fractures	1 (14.29)	
Degenerative changes	0 (0.0)	
Loss of lordosis	0 (0.0)	
Subluxation	0 (0.0)	
Others	0 (0.0)	
CT cervical spine	GCS <14	
Normal	29 (87.88)	0.609
Fractures	2 (6.06)	
Degenerative changes	0 (0.0)	
Loss of lordosis	1 (3.03)	
Subluxation	0 (0.0)	
Others	1 (3.03)	
Distracting injury	Positive CT cervical spine	
Penetrating injury	4 (30.77)	<0.001
Evidence of spinal cord injury	7 (53.85)	
Open or depressed skull fracture	1 (7.69)	
Two or more long bone fractures	0 (0.0)	
Crush or degloving injury	0 (0.0)	
Pelvic fracture	0 (0.0)	
Flail chest	1 (7.69)	
Cervical tenderness	GCS <14	
Yes	6 (6.82)	0.449
No	82 (93.18)	
Cervical tenderness	Positive CT cervical spine	
Yes	11 (47.83)	<0.001
No	12 (52.17)	
FAST	SBP<90	
Positive	1 (5.56)	0.008
FAST	Positive CT cervical spine	
Positive	1 (5.56)	0.468
Disposition care	GCS<14	
Ward	12 (13.64)	<0.001
SCU	46 (52.27)	
ICU	30 (34.09)	
Disposition care	SBP <90	
Ward	7 (38.89)	0.609
SCU	7 (38.89)	
ICU	4 (22.22)	
